# Assessment of Metformin as an Additional Treatment to Therapeutic Lifestyle Changes in Pediatric Patients with Metabolic Syndrome

**DOI:** 10.1155/2012/961410

**Published:** 2012-07-04

**Authors:** Rebecca M. Raub, Stanley J. Goldberg

**Affiliations:** College of Medicine, University of Arizona, 1501 N. Campbell Avenue, Tucson, AZ 85724, USA

## Abstract

*Objective*. To assess the effectiveness of metformin and therapeutic lifestyle changes (TLCs) in a clinical setting, compared to TLC alone in adolescents with metabolic syndrome (MS). *Methodology*. This study was a retrospective trial consisting of 60 patients, aged 8–18 years, who were treated for MS at an outpatient clinic. Two groups were formed: the metformin group (M group) and the control group (C group). The M group had been given metformin along with TLC, and the C group had been given TLC alone. Several outcome measures were obtained; the main outcome measure was measuring the change in percentile and z-score of weight and BMI. *Results*. There were no significant differences between the two groups at the conclusion of the study, except for height percentile (*P* = 0.02) and z-score (*P* = 0.03). Both groups showed promising significant intragroup decreases in weight z-score but BMI percentile and z-score were only significantly decreased in the M group. *Conclusion*. Metformin at an average dose of 1033 mg, when added to TLC, did not show any clinically important efficacy compared to TLC alone in a pediatric population with MS. However, both groups made significant changes in a positive direction, which may be solely due to TLC.

## 1. Introduction

During the last 30 years, childhood obesity rates have more than tripled in the United States [1]. The National Health and Nutrition Examination Survey (NHANES) from 2007-2008 estimated that 16.9% of children from ages of 2- to 19-years old were obese [1]. In 12–19 year olds the obesity rate has increased from 5.0% in the 1976–1980 NHANES survey to 18.1% in 2007-2008 [1]. Further, a strong correlation between childhood obesity and adult obesity has been found and an increasing need to intervene at a younger age may be important [2]. Despite available data in this field, no FDA approved medications that specifically target weight loss, are available for pediatric patients. 

The most predictive factors for obesity, cardiovascular disease, and diabetes have been defined in the criteria for metabolic syndrome (MS) [3]. These risk factors include: hypertension, glucose intolerance, high triglycerides, low HDL-cholesterol concentrations, and elevated waist circumference [3]. Outcome measures based on these criteria constitute a way to assess the effectiveness of a treatment plan. MS is possibly reversible and early intervention might prevent progression to a more serious illness.

One therapy of particular interest is the medication metformin, which is currently approved for use in type 2 diabetes mellitus. Metformin increases insulin sensitivity, and may assist with glycemic control, dyslipidemia, and diastolic blood pressure [4]. Further, metformin may decrease hyperinsulinemia, which in turn may reduce hunger [5]. Metformin has shown promising effects in several randomized pediatric controlled trials [6–10] but further research is needed to determine (1) if the benefits outweigh the side effects and (2) if metformin has a use in a clinical pediatric outpatient setting, outside of a tightly controlled clinical trial environment.

An observation of NHANES in 2007-2008 was the difference among ethnicities pertaining to increased obesity rate [1]. The increase in obesity over the last 30 years has particularly affected Mexican-American and non-Hispanic Black boys and girls as compared to non-Hispanic White boys and girls [1]. After reviewing the studies performed with metformin in children, most of the studies had a non-Hispanic White majority of participants [6–10]. 

This study investigates the results of treatment with TLC alone, compared to TLC coupled with metformin, in pediatric patients with MS in a predominantly Hispanic population. Our specific aim was to determine in a free-living clinical outpatient setting if metformin plus TLC would give additional reduction in weight and BMI percentile or z-score in pediatric patients with MS as compared to TLC alone in a similar control population. 

## 2. Methodology

The institutional review board of the University of Arizona approved this study. Study participants were retrospectively selected from the electronic medical record database for patients followed in our lipidology clinic between 2006 and 2011. Their selection was based on criteria for a diagnosis of MS and their age being between 8–18 years. No universally accepted criteria are available for diagnosis of pediatric MS. For the purpose of this study, diagnosis of MS was determined based on the presence of 3 or more of the following 5 factors: (1) blood pressure in the 90th percentile or above for age and gender [11], (2) triglycerides in the 90th percentile or above for age and gender [12], (3) HDL in the 10th percentile or below for age and gender [12], (4) a waist circumference in the 75th percentile of above for age and gender [13], and (5) evidence of impaired glucose tolerance or hyperinsulinemia. The latter criteria for our study required fasting glucose >100 mg/dL, fasting insulin > upper limit of normal, or definite evidence of acanthosis nigricans. Patients treated with statins, niacin, fibric acids, bile acids sequestrants, ezetimibe, sulfonylureas, or insulin were disqualified from the study. All patients with type I and type 2 diabetes were excluded. Patients who had a history of treatment with antihypertensive drugs were allowed to remain in the study as long as they still had 3 of the 5 requirements for MS. All patients were initially given detailed uniform instructions, both orally by the treating physician and in written material, regarding TLC for diet and exercise. Compliance with instructions was discussed and oral instructions were repeated during each subsequent visit by the physician as appropriate to the patient in the language of their choice (Spanish or English). Instructions included information regarding substituting an approximate 400 Kcal lunch brought from home instead of a much higher caloric school lunch, normal portion size, avoidance or marked limitation of caloric beverages, and inclusion of additional fruits and vegetables. Sweets and sugared cereals and other high sugar content foods were discouraged. Whole grain products were recommended. Fast food consumption was discouraged or better choices at fast food restaurants were recommended. Exercise was recommended for 30 minutes daily and brisk walking was emphasized. Ancillary personnel such as dieticians, exercise physiologists, pharmacists, and other personnel used in clinical trials were not included in counseling patients. New patient visits with the treating physician were 45 minutes in duration, and follow-up visits were 30 minutes. A single physician treated all patients.

From the patients diagnosed with MS, two groups were formed. Group C, the control group, received only treatment with TLC. Group M patients received treatment with identical TLC and the addition of metformin therapy. Use of metformin for patients to assist with MS treatment was inspired by prior studies [8, 10]. Metformin in our patients was started for clinical indication only and without any plan for a future report. However, it was not used for all MS patients and we elected to use it only for alternating patients. Those in the M group were advised to take a multivitamin to provide vitamin B12. Metformin 500 mg twice a day was utilized and occasional patients were directed to take a higher dose. Potential adverse effects of metformin were explained and patients were directed to contact the physician if problems occurred. Initial data were collected from the time patients were first diagnosed with MS for the C group, or for the visit where they were first prescribed metformin for the M group. This initial data was compared to their last clinic visit. Thus not all patients who were recommended to take metformin were included in the final analysis and not all patients who were recommended TLC alone were included.

Each subject in the group that took metformin was matched, based on age, BMI z-score, and gender, with a TLC patient. The paired subjects at the start had to be within a year of age of each other, of the same gender and within 0.25 z-score for BMI. All participants that did not match to a member from the opposite group were eliminated from the study.

When they first started taking metformin for the M group, and compared to their last visit at the clinic. Information gathered from both groups included: age, gender, weight, height, presence of significant acanthosis, the percentage of body fat measured by caliper [14], waist circumference, fasting glucose, fasting insulin, blood pressure, HDL, LDL, triglycerides, and liver enzymes (AST, ALT). If a patient taking metformin was prescribed a secondary medication that could alter their results, data were collected from the time they first started taking metformin until the time before they first started taking their additional medication. 

Results from the data collection for the 2 groups were compared using a paired *t*-test. Intragroup changes between the first visit and end visit were also compared using a paired *t*-test. Because of changes with growth and age, LDL, HDL, triglyercides, blood pressure, waist circumference, weight, height, and BMI were all converted to percentiles for age to standardize the values. Additionally, z-scores were analyzed for weight, height, and BMI [15].

## 3. Results

From a database of 241 possible subjects, 102 subjects met the requirements for MS and did not meet any of the exclusion criteria, and 46 of them took metformin. From the 46 participants in the M group and the 56 participants in the C group, only 30 from each group were comparable using the matching criteria described in the methodology section (Table 1). The alternation of patients for metformin + TLC and TLC alone was imperfect since some patients in each group did not desire followup or come to further visits. Thus not all patients who were recommended to take metformin were included in the final analysis and not all patients who were recommended TLC alone were included. The ethnicity of both groups was 76.7% Hispanic. The M group had 23.3% non-Hispanic Caucasian participants and the C group had 20% non-Hispanic Caucasian and 3.3% non-Hispanic Black participants. The difference in duration between the groups was significant (*P* = 0.04) (Table 1).

The M group took an average dose of 1033 mg/day of metformin with the median dose of 1000 mg/day. At this dosage, our M population did not report adverse effects when specifically asked. No residual pill counting was employed to test adherence to the medication. Patients affirmed at clinic visits that they were taking the medications. Their prescriptions were refilled at their local pharmacies. However, this did not absolutely guarantee compliance. 

TLC were reviewed at each clinic visit and patients reported usually taking lunch from home to school 4 out of 5 days/week, markedly reducing the volume of caloric beverages, and decreasing portions sizes but usually not to the recommended size of the palm of their hand. Fast food frequency was also reported to be decreased and better choices were common. Exercise was reported to be substantially increased. Walking for a sustained 30 minutes was usually reported at 5 days/week but not the daily exercise as recommended. 

 Measures in both of the groups were compared at the beginning of the study and at the end of the study (Table 2). The only significant difference between measures at the start of the study for the M and C groups was duration of treatment (*P* = 0.04), height measured in percentile (*P* = 0.046) and z-score (*P* = 0.04), and systolic blood pressure (*P* = 0.01).

At the conclusion of the study, the only significant final value comparing the 2 groups was the difference of height between the two groups, which was measured using percentile (*P* = 0.02) and z-score (*P* = 0.03). Lipids (LDL, HDL, and triglycerides) showed no significant change when comparing the M and C groups. AST, ALT, fasting insulin, and fasting glucose were not significantly different. Weight, systolic blood pressure, diastolic blood pressure, waist circumference, the percentage of body fat, BMI percentile, and BMI z-score also showed no significant difference between the two groups at the end of the study (Table 2). 

Intragroup changes from start to end of the study duration were evaluated similarly (Table 3). Despite the fact that there was little change when comparing the 2 groups at the end of the study, both of the groups showed significant intragroup changes in many of the parameters. The M group showed a significant decrease in LDL percentile (*P* = 0.01), a decrease in weight z-score (*P* < 0.004), an increase in height measured in centimeters (*P* < 0.004), a decrease in height percentile (*P* < 0.004), a decrease in height z-score (*P* < 0.004), a decrease in glucose (*P* = 0.02), a decrease in systolic blood pressure (*P* = 0.04), a decrease in BMI percentile (*P* = 0.04), and a decrease in BMI z-score (*P* < 0.004).

The C group showed a significant decrease in AST (*P* = 0.03), a decrease in weight in kilograms (*P* = 0.02), a decrease in weight z-score (*P* = 0.02), an increase in height in centimeters (*P* < 0.004), a decrease in height percentile (*P* < 0.004), a decrease in height z-score (*P* < 0.004), an increase in systolic blood pressure (*P* = 0.050), a decrease in the percentage of body fat (*P* = 0.02), and an increase in BMI (*P* < 0.004). All other values in both of the groups changed in an expected direction but not significantly.

## 4. Discussion

The aim of this study was to determine the effectiveness of metformin plus TLC to treat MS as compared to TLC alone. Results show that at a median dose of 1000 mg/dL of metformin used in combination with TLC produced no significant improvement in outcome as compared to TLC alone for the primary endpoints of weight and BMI. 

Previous research in this area has shown results varying from significant [8], modest [9], small [6], or no effect on overall weight loss [7] and BMI in adolescents taking metformin for MS symptoms. Since most of the other research in this field contains double blind clinical trials that were rigidly controlled [6–10], this study adds a new perspective on the topic. Other studies required participants to meet monthly with a dietician [8], or attend a set amount of sessions with a trained health specialists [6] to make lifestyle modifications, both of which are expensive and would rarely occur in clinical practice. However, the clinical trials that found benefit from metformin, studied it at a higher dose that was almost double that used in this study [6, 8, 9]. A lower dose for our patients was selected to reduce adverse effects, mainly gastrointestinal effects, which probably allowed our patients to continue to take the medication. It is possible that metformin at a higher dose in the clinical setting could be more beneficial, but would have caused more adverse reaction and probably more medication discontinuance. Further research is needed to determine if an increased dosage and what level of dosage would be beneficial in a clinical situation. 

Conducting a clinical study in a pediatric population undergoing a growth spurt, presents difficulties that are not inherent in an adult population. Specifically, in a growing population we must rely on percentile and z-scores to judge significance of change rather than absolute values as used in an adult study.

 In both groups the average height increased which would be expected yet the z-score and percentile went down. This shows that although the absolute height increased, it did not increase at the same rate as before, perhaps as the result of (1) achieving, or nearly achieving, full stature or (2) slowing height increase as a result of decreasing excess calories.

The difference in the two groups at the beginning of the study was minimal. The only significant differences proved to be height and blood pressure. The difference in blood pressure could be attributed to the imbalance of patients that treated with blood pressure medications at the beginning of the study. Three patients from the C group were on anti-hypertensive therapy compared to zero patients in the M group. All patients still met the requirements stated in the methodology section despite their anti-hypertensive therapy and were therefore included in the study. Height percentile and z-score were also significantly different from the beginning of the study but they remained significant at the end as well.

Intragroup measures showed promising changes in the M group and C group. There were significant decreases in weight z-score in both groups, which may be attributable to TLC alone. BMI percentile and z-score significantly decreased in the M group whereas the C group measures did not reach significance. 

The positive change in the C group, demonstrates the effectiveness of TLC in this population. The use of both oral and written instructions allowed patients to ask questions in the office and have a reminder of the information when they went home. Although all patients received the relatively same information, the approach was personalized and the session consisted of a problem identification interviewing technique rather than a disease-centered approach.

Limitations to this study include the population size of the study, the lack of ethnic diversity of the participants, the lower dose of metformin used, some incomplete laboratory values, and the fact that it was retrospective. The ethnicity was predominantly Hispanic, which decreases the applicability of this study to other ethnic groups. The median dose of metformin used was predominantly 1000 mg/day, with an average dose of 1033 mg/day. A higher dose may have produced different results but would probably have been more poorly tolerated. Since this study was retrospective, the visit frequency was not rigidly controlled and results could not be studied at defined intervals between start and end of the study. 

Despite the limitations, this study demonstrates: (1) that metformin at a median dose of 1000 mg in this clinical setting did not produce a greater decrease in weight and BMI percentile and z-score than TLC alone, (2) that TLC demonstrated modest but significant intragroup weight z-score change in an outpatient pediatric MS population outside of a clinical trial.

## Figures and Tables

**Table 1:** Participant characteristics.

	M group	C group	*P* value
Number of participants	30	30	
Average age (yr)	13.2	13.2	1.0
Gender			
Female %	46.7	46.7	
Male %	53.3	53.3	
Ethnicity			
Hispanic %	76.7	76.7	
Non-hispanic White %	23.3	20	
Non-hispanic Black %	0	3.3	
Treatment duration (days)	303	469	0.04
Avg. metformin dose (mg/daily)	1033.3	0	

**Table 2:** *P* values comparing the difference between the M group and the C group at the initial data collection and at the final data collection.

	Initial *P* value	Final *P* value
LDL (mg/dL)	0.86	0.62
LDL (percentile)	0.69	0.30
HDL (mg/dL)	0.68	0.46
HDL (percentile)	0.76	0.78
TG (mg/dL)	0.81	0.95
TG (percentile)	0.51	0.98
ALT (U/L)	0.31	0.16
AST (U/L)	0.26	0.14
Weight (kg)	0.16	0.63
Weight (percentile)	0.21	0.59
Weight (z-score)	0.08	0.22
Height (cm)	0.17	0.26
Height (percentile)	0.046^∗^	0.02^∗^
Height (z-score)	0.04^∗^	0.03^∗^
Insulin (*μ*U/mL)	0.15	0.30
Glucose (mg/dL)	0.07	0.85
BP-systolic (mmHg)	0.01^∗^	0.83
BP-diastolic (mmHg)	0.48	0.19
BP-systolic (percentile)	0.13	0.36
BP-diastolic (percentile)	0.08	0.40
Waist (in.)	0.14	0.24
Waist (percentile)	1.00	0.73
Body fat (%)	0.73	0.72
BMI (kg/m^2^)	0.29	0.36
BMI (percentile)	0.80	0.51
BMI (z-score)	0.42	0.85

^∗^Reaches statistical significance (*P* value <0.05).

**Table 3:** Intragroup initial and final values, change and *P*-values.

		M group				C group		
	Initial	Final	Change	*P* value	Initial	Final	Change	*P* value
LDL (mg/dL)	111.87	108.23	−3.64	0.35	110.52	112.29	1.77	0.52
LDL (percentile)	73.67	65.77	−7.90	0.01^∗^	71.72	41.79	−29.93	0.85
HDL (mg/dL)	38.73	40.04	1.31	0.18	39.69	152.89	113.20	0.49
HDL (percentile)	14.33	16.30	1.97	0.41	15.52	71.07	55.55	0.77
TG (mg/dL)	182.93	151.63	−31.30	0.053	187.69	17.32	−170.37	0.02^∗^
TG (percentile)	91.00	86.48	−4.52	0.13	88.62	86.61	−2.01	0.15
ALT (U/L)	54.69	34.52	−20.17	0.17	38.53	23.32	−15.21	0.12
AST (U/L)	34.06	28.71	−5.35	0.43	27.60	21.91	−5.69	0.07
Weight (kg)	85.74	85.17	−0.57	0.68	78.63	82.74	4.11	0.02^∗^
Weight (percentile)	97.87	96.67	−1.20	0.11	97.03	95.97	−1.06	0.10
Weight (z-score)	2.43	2.20	−0.23	<0.004^∗^	2.21	2.03	−0.18	0.02^∗^
Height (cm)	162.44	165.87	3.43	<0.004^∗^	157.74	161.31	3.57	<0.004^∗^
Height (percentile)	73.13	69.80	−3.33	<0.004^∗^	58.60	53.13	−5.47	<0.004^∗^
Height (z-score)	0.83	0.69	−0.14	<0.004^∗^	0.28	0.12	−0.16	<0.004^∗^
Insulin (*μ*U/mL)	26.14	21.11	−5.03	0.22	19.63	17.26	−2.37	0.13
Glucose (mg/dL)	92.10	86.40	−5.70	0.02^∗^	87.50	86.94	−0.56	0.70
BP-systolic (mmHg)	125.77	120.87	−4.90	0.04^∗^	115.20	120.17	4.97	0.050^∗^
BP-diastolic (mmHg)	71.07	69.70	−1.37	0.49	72.83	72.80	−0.03	0.99
BP-systolic (percentile)	74.40	70.57	−3.83	0.47	65.63	65.30	−0.33	0.95
BP-diastolic (percentile)	57.13	57.30	0.17	0.96	65.93	61.17	−4.76	0.32
Waist (in.)	42.10	41.30	−0.80	0.62	40.21	39.63	−0.58	0.37
Waist (percentile)	89.50	88.45	−1.05	0.29	89.50	88.00	−1.50	0.08
Body fat (%)	34.95	33.14	−1.81	0.26	34.41	32.48	−1.93	0.03^∗^
BMI (kg/m^2^)	32.07	31.07	−1.00	0.44	30.78	33.64	2.86	<0.004^∗^
BMI (percentile)	97.60	95.87	−1.73	0.04^∗^	97.47	96.90	−0.57	0.26
BMI (z-score)	2.23	2.04	−0.19	<0.004^∗^	2.17	2.06	−0.11	0.08

^∗^Reaches statistical significance (*P* value <0.05).

## References

[1] Ogden C, Carroll M

[2] Morrison JA, Friedman LA, Wang P, Glueck CJ (2008). Metabolic syndrome in childhood predicts adult metabolic syndrome and type 2 diabetes mellitus 25 to 30 years later. *Journal of Pediatrics*.

[4] Saenz A, Fernandez-Esteban I, Mataix A, Ausejo M, Roque M, Moher D (2005). Metformin monotherapy for type 2 diabetes mellitus. *Cochrane Database of Systematic Reviews*.

[5] Han JC, Rutledge MS, Kozlosky M (2008). Insulin resistance, hyperinsulinemia, and energy intake in overweight children. *Journal of Pediatrics*.

[6] Wilson DM, Abrams SH, Aye T (2010). Metformin extended release treatment of adolescent obesity: a 48-week randomized, double-blind, placebo-controlled trial with 48-week follow-up. *Archives of Pediatrics and Adolescent Medicine*.

[7] Love-Osborne K, Sheeder J, Zeitler P (2008). Addition of metformin to a lifestyle modification program in adolescents with insulin resistance. *Journal of Pediatrics*.

[8] Srinivasan S, Ambler GR, Baur LA (2006). Randomized, controlled trial of metformin for obesity and insulin resistance in children and adolescents: improvement in body composition and fasting insulin. *Journal of Clinical Endocrinology and Metabolism*.

[9] Yanovski JA, Krakoff J, Salaita CG (2011). Effects of metformin on body weight and body composition in obese insulin-resistant children: a randomized clinical trial. *Diabetes*.

[10] Freemark M, Bursey D (2001). The effects of metformin on body mass index and glucose tolerance in obese adolescents with fasting hyperinsulinemia and a family history of type 2 diabetes. *Pediatrics*.

[11] Falkner B, Daniels SR, Flynn JT (2004). The fourth report on the diagnosis, evaluation, and treatment of high blood pressure in children and adolescents. *Pediatrics*.

[12] Daniels SR, Greer FR (2008). Lipid screening and cardiovascular health in childhood. *Pediatrics*.

[13] Fernandez JR, Redden DT, Pietrobelli A, Allison DB (2004). Waist circumference percentiles in nationally representative samples of African-American, European-American, and Mexican-American children and adolescents. *Journal of Pediatrics*.

[14] Durnin JVGA, Womersley J (1974). Body fat assessed from total body density and its estimation from skinfold thickness: measurements on 481 men and women aged from 16 to 72 years. *British Journal of Nutrition*.

[15] Centers of Disease Control and Prevention http://www.cdc.gov/growthcharts/.

